# A More Homogeneous Phenotype in Parkinson's Disease Related to R1441G Mutation in the LRRK2 Gene

**DOI:** 10.3389/fneur.2021.635396

**Published:** 2021-03-02

**Authors:** Ana Vinagre-Aragón, David Campo-Caballero, Elisabet Mondragón-Rezola, Lara Pardina-Vilella, Haizea Hernandez Eguiazu, Ana Gorostidi, Ioana Croitoru, Alberto Bergareche, Javier Ruiz-Martinez

**Affiliations:** ^1^Department of Neurology, Hospital Universitario Donostia, San Sebastián, Spain; ^2^Neuroscience Area, Biodonostia Health Research Institute, San Sebastián, Spain; ^3^Centro de Investigación Biomédica en Red para Enfermedades Neurodegenerativas (CIBERNED), Carlos III Health Institute, Madrid, Spain

**Keywords:** R1441G, LRRK2, phenotype, Parkinson's disease, progression

## Abstract

Parkinson's disease (PD) is characterized by a great clinical heterogeneity. Nevertheless, the biological drivers of this heterogeneity have not been completely elucidated and are likely to be complex, arising from interactions between genetic, epigenetic, and environmental factors. Despite this heterogeneity, the clinical patterns of monogenic forms of PD have usually maintained a good clinical correlation with each mutation once a sufficient number of patients have been studied. Mutations in LRRK2 are the most commonly known genetic cause of autosomal dominant PD known to date. Furthermore, recent genome-wide association studies have revealed variations in LRRK2 as significant risk factors also for the development of sporadic PD. The LRRK2-R1441G mutation is especially frequent in the population of Basque ascent based on a possible founder effect, being responsible for almost 50% of cases of familial PD in our region, with a high penetrance. Curiously, Lewy bodies, considered the neuropathological hallmark of PD, are absent in a significant subset of LRRK2-PD cases. Indeed, these cases appear to be associated with a less aggressive primarily pure motor phenotype. The aim of our research is to examine the clinical phenotype of R1441G-PD patients, more homogeneous when we compare it with sporadic PD patients or with patients carrying other LRRK2 mutations, and reflect on the value of the observed correlation in the genetic forms of PD. The clinical heterogeneity of PD leads us to think that there may be as many different diseases as the number of people affected. Undoubtedly, genetics constitutes a relevant key player, as it may significantly influence the phenotype, with differences according to the mutation within the same gene, and not only in familial PD but also in sporadic forms. Thus, extending our knowledge regarding genetic forms of PD implies an expansion of knowledge regarding sporadic forms, and this may be relevant due to the future therapeutic implications of all forms of PD.

## Introduction

Parkinson's disease (PD) is the second most common neurodegenerative disorder, after Alzheimer's disease. The worldwide incidence estimates range from 5 to more than 35 new cases per 100,000 individuals yearly (1). This relatively wide range is probably due to differences in study methodologies or in the demographic characteristics of the populations studied. The overall prevalence in the general population is estimated at 0.3% but rises sharply with age to more than 3% in those >80 years of age (2). PD is clinically defined by the presence of bradykinesia and at least one additional cardinal motor feature (rigidity or rest tremor), as well as additional supporting and exclusionary criteria (3). In addition, the disease is associated with a wide spectrum of non-motor symptoms (NMS). These include cognitive impairment, disorders of sleep–wake cycle regulation, autonomic dysfunction, and mood disorders, as well as sensory symptoms (most prominently hyposmia and pain). Some of these NMS can predate the onset of classic motor symptoms by years or even decades (4). PD is mainly characterized by a selective slow and progressive degeneration of dopaminergic neurons in the substantia nigra. Nevertheless, non-dopaminergic neurons are also known to degenerate in PD. In fact, the pathological process also affects even neurons outside the central nervous system (CNS), such as those in the mesenteric system or olfactory bulb. In addition to neuronal loss, this disorder is pathologically characterized by the presence of abnormal deposition of α-synuclein (αsyn) in the cytoplasm of certain neurons (Lewy bodies) in several different brain regions. However, Lewy bodies are not specific to the diagnosis of PD and PD can be diagnosed even in the absence of Lewy body pathology (5).

Despite intensive research, the etiopathogenesis of PD remains largely unknown, considering currently that there is no single cause and that there are multiple factors that play a role in its development, especially genetic, environmental, and also epigenetic factors. The genetic forms of PD justify only around 10–15% of cases of PD and are derived from mutations in genes that are involved in different cellular processes. The functional characterization of these genes has enabled the scientific community to understand a series of basic cellular mechanisms that intervene also in the sporadic form of PD (sPD), although its hierarchy in the latter is still unknown. Among these cellular mechanisms are those related to defects in folding, aggregation, and phosphorylation of αsyn, abnormalities in intracellular vesicular trafficking, neuroinflammation, and oxidative stress derived from mitochondrial dysfunction (6). All these proposed pathways might be involved, are non-mutually exclusive, and all are susceptible to be pharmacologically manipulated to try to intervene early in the etiopathogenesis of the disease.

The most frequent forms of familial PD transmitted with dominant inheritance are the forms linked to mutations in leucine-rich repeat kinase 2 (LRRK2) gene. LRRK2 is a large, multidomain protein containing two catalytic domains: a Ras of complex proteins (Roc) G-domain and a kinase domain. Multiple variants of this gene have been described, yet only 8 have been proved to be pathogenic (N1437H, R1441 G/H/C, Y1699C, I2012T, G2019S, and I2020T) (7). Mutations have been identified in both catalytic domains and in several of its multiple putative regulatory domains (8, 9). Moreover, recent genome-wide association studies (GWAS) have revealed variations in LRRK2 as significant risk factors also for the development of sPD (10, 11). Although definite molecular consequences of mutations in LRRK2 have not been fully elucidated, different studies indicate that mutations occurring in the LRRK2 gene are associated with an increase in kinase activity (12). Thus, the fact that these mutations result in hyperactivation of the LRRK2 kinase has broadened disease-modifying treatment's horizon and, indeed, LRRK2 kinase inhibitors are being developed and tested, suggesting that subjects with LRRK2 mutations may be one of the first precision medicine cohorts for PD.

**Figure 1:**
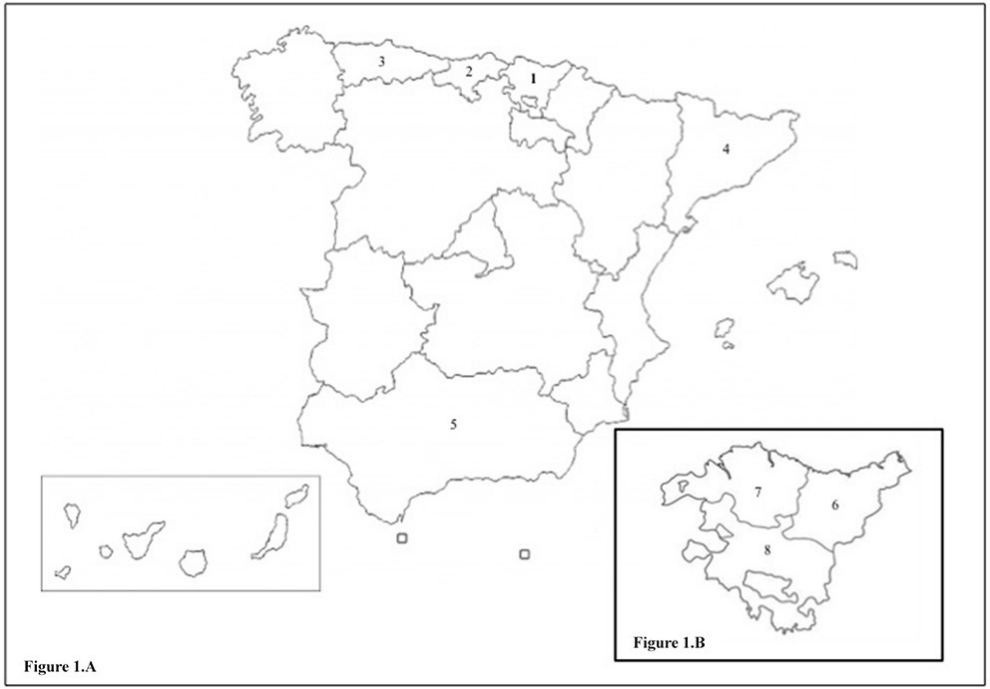
**(A)** Map of Spain. 1. Basque Country. 2. Cantabria. 3. Asturias. 4. Cataluna. 5. Andalucia. **(B)** Enlarged map of the Basque Country with its provinces. 6. Gipuzkoa. 7. Bizkaia. 8. Araba.

The R1441G mutation is especially frequent in the Basque population, especially in the region of Gipuzkoa, based on a possible founder effect due to the presence of a common haplotype (13). The prevalence of this mutation is 46% in familial forms of PD and 2.5% in sporadic forms in patients of Basque origin in Gipuzkoa (14). The R1441G mutation has also been identified in Bizkaia (15) and in other regions near the Basque Country with a prevalence of 2.2% in Asturias (16), 1% in Cantabria (17), and 0.7% in Cataluña (18) (Figures 1A,B). It has been posited that a “north-south” gradient may exist, since R1441G-PD is significantly less common as we descend in the peninsula, with just isolated cases in Andalucía, which also share the same haplotype. In contrast, this mutation is very uncommon in other European populations, including other regions of Spain, North and South America, and Japan (14, 19–22). Lifetime penetrance of R1441G mutations increases with age, with figures of 12.8% at 65 years, from 50.2% at 70 years, reaching 83.4% at 80 years of age (23).

The main objective of this work is to summarize the results of the most relevant publications related to the clinical phenotype of patients with R1441G-PD. In addition, updated data from the registry of our patient cohort, based on our collaboration in the Parkinson's Progression Markers Initiative (PPMI) and on our daily clinical practice, will be provided.

## Published Clinical Phenotype of the R1441G-PD Patients

### Motor Symptoms

The motor aspects of R1441G-PD seem to be very similar to sPD. The first publications reported an age of onset around 60 years and frequently an asymmetric tremor-dominant phenotype (24, 25). The clinical course was slowly progressive, with an excellent response to dopaminergic treatment. Most of the patients developed motor fluctuations and dyskinesias over the years while dementia was uncommon even 15 years after the onset of the disease (25). In subsequent studies, it was found that asymmetric resting tremor predominated as onset symptom (60%) and that classic motor complications (fluctuations in 63.5%, dyskinesias in 56.5%) appeared at the same time as in sPD (26). Moreover, it was observed that the presence of dystonia throughout the disease stood out in 22.6% of cases predominantly in the lower limbs. In most reported cases, it was a tonic extension of the great toe. In some cases, it was a debut symptom, a characteristic sign and very homogeneous in certain families.

### Cognitive Dysfunction

Cognitive impairment in PD associated with LRRK2 mutations is the most studied NMS, and existing evidence suggests that dementia is uncommon in these patients even one or two decades after the onset of the disease. Initial studies of cognitive dysfunction in patients with LRRK2-PD were based on clinical observations and suggested that dementia might occur less frequently in LRRK2-PD carrying R1441G and G2019S mutations than in sPD (25, 27). Kasten et al. evaluated the presence of dementia in ~65% of patients with LRRK2-PD, finding a prevalence of 11% in these patients. These figures are lower than those observed in sPD (28), where around 25–30% of patients develop dementia (29, 30). Subsequent studies found similar cognitive abnormalities in G2019S-PD and sPD patients, being executive function the most frequently impaired domain (31–33). Our group performed a study to examine the cognitive status of R1441G-PD and compared this to that of sPD. A comprehensive cognitive assessment was performed using an extensive neuropsychological battery in order to evaluate the different cognitive domains. No differences were found in neuropsychological performance of R1441G-PD and sPD patients (34). The prevalence of mild cognitive impairment (PD-MCI) was 30% in both groups, with no differences in the number and type of domains impaired. Executive function, memory, and attention were the most frequently affected domains. Although the difference was not statistically significant, the prevalence of dementia was higher in the sPD group (27 vs. 13%). These results were in line with prior studies, which suggested that impairment of executive function and attention was frequent in LRRK2-PD patients, whereas dementia was not so common (35). Somme et al. examined cognition and psychiatric symptoms in 27 patients with LRRK2-PD (12 G2019S and 15 R1441G) and 27 patients with sPD. LRRK2-PD patients exhibited less frequently subjective cognitive complaints and mild cognitive impairment or dementia (36). More studies are needed, but what has been published to date suggests that cognitive impairment is less frequent in LRRK2-PD, more specifically in PD associated with the R1441G mutation.

A study of the prodromal phase of PD with asymptomatic, non-manifesting carriers (NMC) of LRRK2 mutations has recently been conducted. G2019S carriers scored higher in motor scores and had lower radioligand uptake compared to non-carriers, but no differences in NMS scores were observed. In contrast, R1441G carriers scored higher in motor scores, had lower radio ligand uptake, and had higher scores in depression, dysautonomia, as well as REM Sleep Behavior Disorder Screening Questionnaire (RBDSQ) scores, but had better cognition scores than non-carriers (37).

### Affective and Neuropsychiatric Symptoms

In the cohort of patients with G2019S-PD studied by Goldwurm et al. it was found that the majority of subjects (14/16) had affective and behavioral alterations on the NPI-12 scale, mainly depression, anxiety, irritability, and hallucinations (32). According to the previously mentioned review carried out in patients with genetic PD (28), the prevalence of depression was 30% in LRRK2-PD patients. In contrast, in other studies prevalence reached 50–65% (31, 32). Compared with sPD, different results have hitherto been reported. In some studies, no differences have been noted (33, 38–41), while in others fewer depressive symptoms in patients with G2019S-PD were observed (42). With regard to anxiety, Belarbi et al. described a percentage of anxiety (measured through the NPI) of 69%. Compared to sPD, no significant differences were observed (56%) (31). In this study, the authors also found a high frequency of apathy (56 vs. 35%), irritability (34 vs. 20%), sleep disturbances (65 vs. 39%), and hallucinations (26 vs. 6%) compared with patients without mutation, with significant differences in the case of depression and anxiety. Somme et al. reported a lower prevalence of hallucinations and apathy in LRRK2-PD patients (R1441G and G2019S) compared to patients with sPD (36). In contrast, Gaig et al. found no differences between G2019S-PD and sPD in terms of hallucinations, anxiety, and depression (40). A link between bipolar disorder and LRRK2 gene has also been suggested (33). Our group evaluated affective symptoms in R1441G-PD. Thirty patients with R1441G-PD were compared with 30 sPD. The mean scores in the depression and anxiety scales were similar in both groups. Depressive symptoms were detected in 31.8% of R1441G-PD and 25% of sPD patients, and anxiety symptoms were evident in 4.5 and 15%, respectively (34). No further studies have hitherto been conducted to assess the presence of depressive symptoms and anxiety specifically in R1441G-PD.

### Hyposmia

Hyposmia is one of the most common and best-characterized NMS and is often one of the earliest prodromal features to manifest. Due to the fact that prior existent evidence suggested that odor identification appeared to be distinctly affected in LRRK2-PD patients (27, 43, 44), our group performed a study to assess olfactory function in PD patients using the Brief Smell Identification Test (B-SIT) and compared carriers of the G2019S and R1441G mutations in LRRK2 with non-carriers (45). A total of 190 PD patients were assessed, consisting of 146 non-carriers and 44 carriers of a mutation in LRRK2, 39 of which were R1441G mutations and 5 were G2019S mutations. Olfactory dysfunction was distributed distinctly between groups. Out of 44 LRRK2 mutation carriers, only 16 (36%) exhibited hyposmia. In contrast, hyposmia was evident in 110 of the 146 non-carriers (75%). Despite the fact that similar results were found in both mutations in this study, the small sample of patients carrying the G2019S mutation hindered reaching a definitive conclusion regarding this mutation. Nevertheless, according to the results of the study, a normal olfactory test result in a patient with typical PD may increase the probability that the patient is a LRRK2 mutation carrier, specifically the R1441G mutation.

### Sympathetic Dysfunction

The sympathetic function can be evaluated by cardiac scintigraphy, measuring the uptake of 123I-metaiodobenzylguanidine (MIBG). Our group evaluated a total of 90 patients by cardiac MIBG scintigraphy, including 27 carriers of LRRK2 mutations (23 with the R1441G mutation and 4 with the G2019S mutation) and 63 non-carriers (45). Sixty-six percent of LRRK2 mutation carriers had low early MIBG uptake, compared to 86% of non-carriers (*P* = 0.048). Similarly, the heart/mediastinum ratio in delayed MIBG images appeared to differ between these groups of patients with PD, even though these results did not reach statistical significance.

### Sleep Disorders

Sleep disorders such as insomnia, excessive daytime sleepiness (EDS), and REM Sleep Behavior Disorder (RBD) are common in sPD. In fact, RBD, and possibly EDS, may antedate the onset of parkinsonism in sPD. Iranzo et al. assessed sleep in 18 LRRK2-PD (17 carrying G2019S and one R1441G mutations), 17 NMC (11 G2019S, three R1441G, three R1441C), 14 non-manifesting non-carriers (NMNC), and 19 unrelated sPD through a comprehensive interview conducted by sleep specialists, validated sleep scales and questionnaires, and video-polysomnography followed by multiple sleep latency test (MSLT). They observed that sleep complaints were frequent in LRRK2-PD and showed a pattern that when compared to sPD was characterized by more frequent sleep onset insomnia, similar EDS, and less prominent RBD. Thus, unlike in sPD, RBD and EDS seemed to be not suitable markers of the prodromal stage of LRRK2-PD (46). However, further studies are needed to asses sleep disorders specifically in R1441G-PD.

## Our Series of R1441G-PD Patients and Non-Manifesting Mutation Carriers

In the last 25 years, we have been closely monitoring familial forms of PD in the Movement Disorders Unit of Donostia University Hospital (San Sebastián, Gipuzkoa, Spain). A total of 251 LRRK2 mutation carriers have been followed up. Sixty-six of them carry the G2019S mutation (46 PD patients and 20 NMC). Regarding the R1441G mutation, we have followed up 100 R1441G-PD patients, as well as more than 200 of their asymptomatic relatives, of which 85 NMC have been registered. Both patients and their families have collaborated altruistically in multiple projects and consortia. In this article, we would like to show the current general characteristics of this cohort, and some data about its follow-up obtained in the context of the Parkinson's Progression Markers Initiative (PPMI) as well as our general daily practice.

### Characteristics of the R1441G Series of Patients

The general characteristics and the motor phenotype of R1441G-PD patients remain very similar to those described in 2012 by Ruiz-Martinez J. in his doctoral thesis (Table 1) (26). The 100 patients currently included in the database have the diagnosis of PD according to the Gelb and Brain Bank clinical criteria. Ninety-nine patients are heterozygous for the R1441G mutation, and one is homozygous. Ninety-three percent have positive family history, mostly first-order (80%). All these patients belong to a total of 49 families. We have been able to follow-up patients from three generations. Some of these families have up to 20 members suffering from PD. We have only had one case of phenocopy. Eighty-one percent are of Basque origin based on the presence of at least their first two surnames with this root.

**Table 1:** Characteristics of the R1441G-PD patients in 2012 and updated date about the current cohort.

	**2012 Ruiz-Martínez J**.	**2020 Vinagre-Aragón A**.
Basque origin	94%	81%
PD family history	91%	80%
Homozygous	0	1%
Phenocopy	0	1%
Gender (male/female)	48.5%	51%
Mean age (years)	74.6	77.9
Mean age at onset (years)	61.8	62.7
Mean disease duration (years)	12.7	14.9
Levodopa response	100%	100%
Equivalent levodopa (mg/day)	875	767.1
Advanced therapies - Deep Brain Stimulation (DBS) - Continuous apomorphine infusion (CAI) - Continuous intrajejunal infusion of levodopa/carbidopa gel (CIILG)	1 DBS	5 DBS, 3 CAI, 1CIILG
H&Y	2.91	2.71
Motor phenotype		
- Tremor	60%	55.36%
- Rigid-akinetic syndrome	27.7%	27.69%
- Mixt	7.7%	3.11%
- Gait predominant	4.6%	13.84%
Neuropathological study	2 (no αsyn aggregates)	5 (no αsyn aggregates)

Regarding the distribution by sex, 51 are women and 49 are men. In this series, the current mean age of the patients (*n* = 95) is 77.95 years (40-96), the mean age of disease onset (*n* = 90) is 62.71 years (35-81), and the mean time of duration of disease (*n* = 89) is 14.9 years (4-33).

The clinical phenotype at disease onset was analyzed in 65 patients, and tremor (55.36%) predominates clearly over rigid-akinetic syndrome (27.69%) and gait disorder (13.84%). The current mean H&Y stage (*n* = 0) is 2.71, with patients predominating in stages 3 (45.71%) and 2 (24.2%). Regarding NMS, there are data available published in 2012 by Ruiz-Martinez J. in his Doctoral Thesis (26). The frequency of NMS was analyzed in 68 R1441G-PD patients and compared with 28 G2019S-PD patients. At the time of the evaluation, 35.9% had signs of cognitive impairment, 4.8% suffered from hallucinations, and 31.3% had depressive symptoms. Other NMS were observed with the following frequency: constipation 30.6%, orthostatic hypotension 11.3%, RBD 11.5%, and EDS 4.8%. No case associated with restless leg syndrome was reported. When comparing R1441G-PD and G2019S-PD, there were only significant differences in hallucinations (4.8 vs. 29.2%) and urinary symptoms (19.4 vs. 43.5%).

The equivalent dose of levodopa (L-dopa) is 767.1 mg/day. Regarding advanced therapies, 5 patients have undergone deep-brain stimulation (DBS), two associate continuous infusion of apomorphine, and one patient is on intrajejunal duodopa infusion. All the patients that underwent DBS evolved well. In all of them, the fluctuations and dyskinesia improved significantly, and none of them developed complications. In 3 of them, a 50% reduction in L-dopa was achieved. Freezing was the symptom that improved the least, as is to be expected in PD patients in general.

Within the subgroup of patients with an earlier age of onset, there is a patient who is also a carrier of a mutation that justifies cerebral calcifications observed in him and his relatives. Another patient associates a mutation in Parkin gene, and another suffers from Down syndrome. The Parkin/LRRK2 double-mutation case has a homozygous deletion in Parkin considered pathogenic, and the phenotype of a very tremulous early onset coincides with the classic phenotype of Parkin-associated PD.

In this historical series, 42 patients have already died at a mean age of 82.02 years (63–93). The age at disease onset in these cases was 65.79 years (4–80), and they died after a mean duration of disease of 16.02 years (5–27). It was possible to perform an autopsy in 5 of these patients. Curiously, Lewy bodies and Lewy neurites were absent in all of them in the neuropathological study (also in the double mutation case).

### Non-manifesting Carriers of the R1441G Mutation Follow-Up

In a study carried out by our group on asymptomatic relatives with the R1441G mutation, there was evidence of dopaminergic nigrostriatal denervation in R1441G mutation carriers and it was associated with a decrease in the performance of complex motor tests. These tests could be early indicators of ongoing dopamine deficit in this group at risk for PD (47). It should be pointed out that to date, we still are in touch with 89 NMC (49 women and 40 men) with a mean age of 52.4 years (38-73).

### PPMI Cohort

As previously mentioned, our hospital is one of the clinical sites in which the PPMI takes place with a total of 28 participants carrying the R1441G mutation involved (14 PD patients and 14 asymptomatic mutation carriers). Taking part in this study has enabled us to closely monitor these subjects during a 6-year follow-up period.

**Figure 2:**
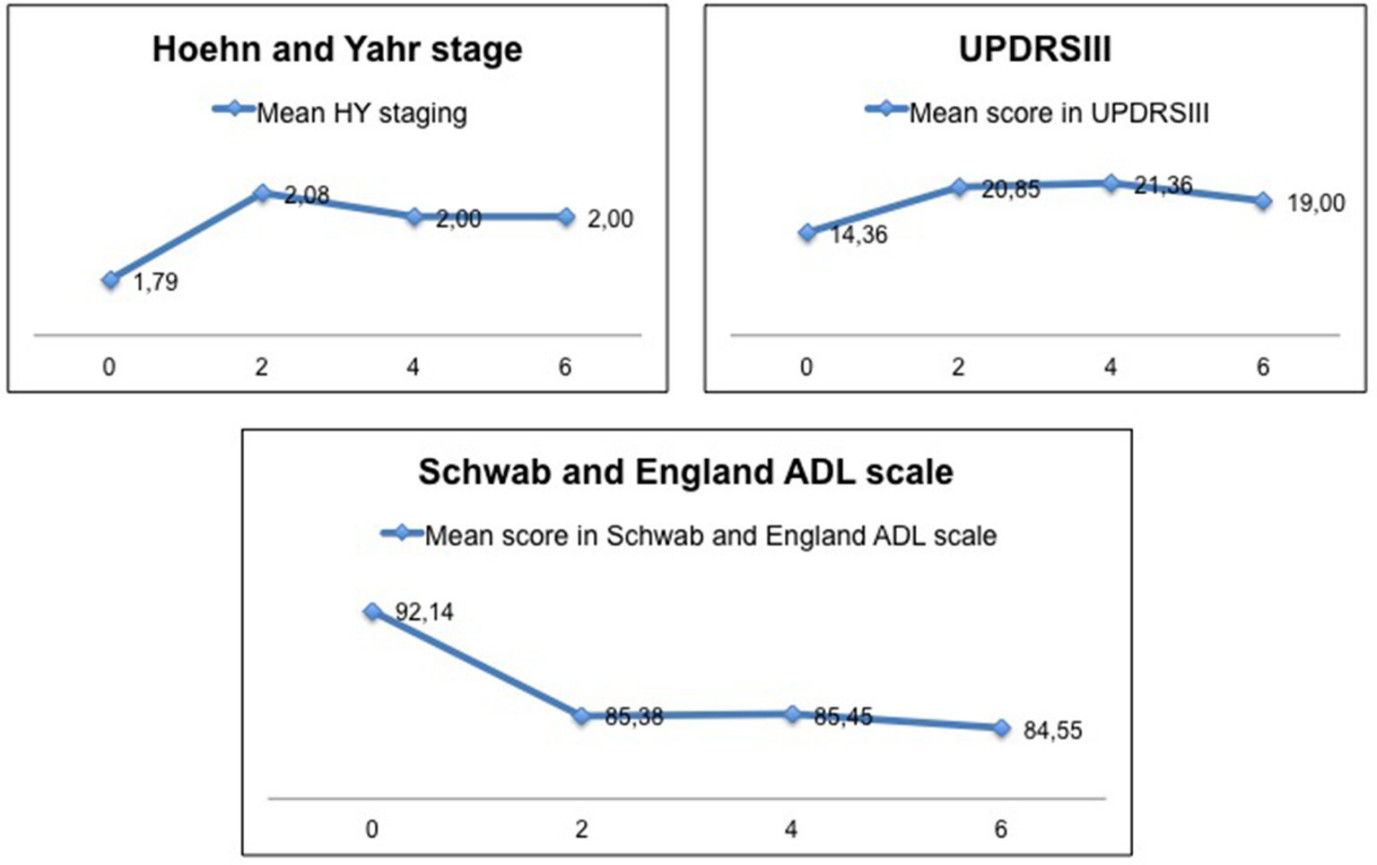
Motor progression and its impact on activities of daily living in the subgroup of patients of our cohort participating in the PPMI.

The mean age at onset of disease in R1441G-PD patients was 63 (46-80) years. The mean duration of disease when follow-up began was 9 (5–14) years. The PD phenotype at disease onset was tremulous in 70%, with first Parkinson's symptom being leg rest tremor in all these cases. Ninety percent had positive family history. As shown in Figure 2, motor progression was certainly slow, with the mean UPDRSIII at baseline of 14 ± 5 (5–26) and the mean UPDRSIII at year 6 of 16 ± 11 (4–44). Likewise, the mean HY at baseline was 1.79 and 2.00 at year 6 of follow-up. The mean score on the Schwab and England ADL scale at baseline was 92 ± 7 (80–100) and 84 ± 17 (60–100) at year 6. Patients remained cognitively stable during follow-up. The mean score on MOCA at baseline was 23 ± 3 (18-28) and 24 ± 3 (19-29) at year 6.

The mean age of NMC when follow-up began was 59 (51-65). In 40%, basal DaTSCAN was consistent with evidence of dopamine transporter deficit. In the context of PPMI, DaTSCANs were performed in NMC at 2, 4, and 6 years from the start of follow-up, but these data have yet not been published. To date, none of them have manifested symptoms or signs of the disease.

## Discussion

The first descriptions in 2004 of PD associated with the R1441G mutation on a small sample of patients (13, 24, 25) showed motor features very similar to those obtained in the doctoral thesis of Ruiz Martinez J., in 2012 (26) and in the current work, which includes a significantly larger sample. The clinical profile of PD associated with R1441G mutation is very similar to that usually described in sPD. Certain NMS are less common in R1441G-PD, and in addition, these PD patients appear to have also less cognitive impairment. In fact, the latter could be a possible clinical difference between R1441G-PD and sPD patients or patients carrying other mutations in the LRRK2 gene, but further studies with larger samples are needed. However, the fact that the same features are maintained after increasing the sample size and expanding the follow-up period supports the initial hypothesis that R1441G-PD is characterized by a homogeneous clinical phenotype.

A close follow-up of patients and NMC has allowed us to know more accurately the evolution of the disease in these cases and to analyze the role of different clinical markers for the detection of early signs of the disease. There is no doubt that the study and follow-up of NMC is an excellent opportunity for the detection of these markers. The relevance of certain NMS is known due to their early presence before the so-called motor phase of the disease, and therefore the study of these NMS in R1441G mutation carriers allows us to approximate the value of these symptoms as PD predictor markers. However, within NMS, olfactory and sympathetic dysfunction do not seem to be highly represented in R1441G-PD patients. Thus, neither seems to be a marker in NMC. Similarly, RBD and constipation appear to be also less represented in R1441G-PD patients according to previously published data. In contrast, as might be expected in a predominantly motor disease in R1441G-PD patients, the follow-up data of NMC and their correlation with the DaTSCAN (47) indicate that the study of early markers should be aimed at evaluating areas or systems that have shown loss of activity, and in this sense the nigrostriatal dopaminergic pathway stands out as it defines the motor profile of the illness. Thus, the DaTSCAN as well as other tests evaluating the nigrostriatal motor pathway may be more effective in preclinical stages as they evaluate areas where neuronal degeneration has been demonstrated.

In addition, long-term follow-up has enabled to obtain mortality data as well as the performance of neuropathological studies that help to improve knowledge about the etiopathogenesis of the disease. So far, 42 R1441G-PD patients have died. The mean age of death, after more than a mean of 16 years of duration of disease, reaches 82 years. This figure is very similar to the mean age of death expected in the Spanish population, which is around 83 years. This data also indicates that it is a disease with a less aggressive course.

In 5 of the deceased patients, informed consent to perform the neuropathological study was obtained. In these cases, the course of the disease was characterized by a lower frequency of NMS and all of them showed isolated nigral degeneration in the absence of Lewy pathology (48). Takanashi et al. reported the same findings (49). Some of these cases were included in the work published by Kalia et al. (50). They performed a clinical–pathological correlation study in a series of patients with LRRK2-PD and showed that the cases with less accumulation of αsyn corresponded to forms of PD with a pure motor phenotype and less presence of NMS. Unlike in R1441G-PD, G2019S, and I2020T-PD did show greater heterogeneity in the results of the hitherto performed neuropathological studies (5).

It should also be pointed out that one of the recently deceased patients carried also a Parkin mutation. In this specific case, the age of disease onset was early (44 years) and the phenotype was very tremulous coinciding with the classic phenotype of Parkin-associated PD. The course was less aggressive with few NMS. The patient died 22 years after the onset of symptoms due to a lymphoma, and αsyn aggregates were absent in the neuropathological study.

The absence of αsyn aggregates in ours and other descriptions of LRRK2-PD patients can be understood from different perspectives. The surmise reason of this difference may be the location of the mutation in the protein domain. G2019S and I2020T mutations are located in the kinase domain, whereas R1441G/H is located in the Roc domain. The kinase domain is known to be associated with the Rab family of proteins, and therefore, it seems to be more likely related to vesicular trafficking, autophagy, and/or lysosomal dysfunction resulting in a cascade that induces Lewy body pathology (51). Nevertheless, R1441G/H mutation may more directly relate to the dopaminergic neuronal loss. Actually, many patients with R1441G/H pathologically showed isolated nigral degeneration in the absence of Lewy pathology (48, 49). The later histopathological features may constitute a marker of slower neuronal loss, which could justify the less aggressive clinical course observed in these patients. Patients carrying other LRRK2 mutations (G2019S and I2020T) have shown heterogeneity in the results of the performed neuropathological studies, which corresponds to a more variable clinical phenotype. This variability makes us consider that part of the pathophysiological mechanisms considered pivotal in the development of the disease up to now could not play such a central role.

Different physiopathogenic hypotheses have been proposed, based on abnormal aggregation and subsequent deposition of αsyn, with a toxic effect in the areas where it is deposited. In contrast, in R1441G-PD patients where these inclusions appear to be absent, the neuronal death mechanism has to be explained in another way. This plausible different physiopathogenic mechanism seems to affect the nigrostriatal pathway and determine a similar clinical motor phenotype. Nevertheless, it does not seem to affect other areas involved in the pathophysiology of NMS in sPD. Thus, the deposit of αsyn may constitute an epiphenomenon of the toxicity of other abnormal proteins involved in the pathogenesis of neurodegeneration in the context of aging and certain environmental factors.

It is extremely important to know in detail the specific phenotype of each type of PD in order to be able to study early-onset clinical markers and, based on this, to be able to develop disease-modifying therapies. The R1441G mutation has been consistently related to a motor phenotype similar to that seen in sPD over the years of follow-up. Without taking into account other potential neuroimaging or metabolic markers, the lower presence of NMS leads us to design the search for early markers of the disease focused on the nigrostriatal pathway. The homogeneity of these results correlates with a specific neuropathological pattern without αsyn aggregates as in other case descriptions of LRRK2-PD.

## Author Contributions

AV-A: literature review, data processing and analyses, results interpretation, language editing, review, and drafting of the first manuscript. JR-M: conception and article design, literature review, data processing and analyses, results interpretation, and critical revision of the manuscript. All authors contributed to the article and approved the submitted version.

## Conflict of Interest

The authors declare that the research was conducted in the absence of any commercial or financial relationships that could be construed as a potential conflict of interest.
